# Miscellaneous Notices

**Published:** 1849-01

**Authors:** 


### Miscellaneous Notices.

### MARCH, 1849.

*Annual Commencement of the Baltimore College of Dental Surgery, Session* 1848–'49.—The annual commencement for conferring the degree of Doctor of Dental Surgery, was held in the hall of the College building, on the evening of March 1st; the candidates having previously been subjected to a rigid examination by the Faculty, in the presence of an Examining Committee, composed of eminent dentists and physicians, appointed agreeably to a recommendation of the American Medical Association.

At an early hour the hall was crowded, and the exercises commenced with prayer, appropriate to the occasion, by the Rev. Stephen Hill.

The *authority* for conferring the degree was next read by the Dean of the Faculty—after which Doctor Eleazar Parmly, of New York, Provost of the College, arose to confer the degree, previously making, however, a few appropriate prefatory remarks to the graduating class, in reference to the high responsibilities they were about to assume; and that, in view of which, the indispensable necessity of each one cultivating for himself an elevated moral and scientific character, as the strongest guarantees for raising the standard of dental qualifications, as well as of dental skill, in the profession of his choice. We refer to another part of the Journal for the Doctor's remarks.

Before taking his seat, Prof. C. A. Harris called up successively, by name, the graduating class, stating the residence and *thesis* of each, when the Provost, at the same time, handed a diploma to each one respectively, feelingly remarking that it was the just reward severally due their high qualifications and merit.

The Report of the Infirmary came next in order, and was read by Prof. C. O. Cone, giving the practice of the students in this department, and showing the number and kind of dental operations therein performed the details of which are as follows:

Report of the Infirmary and Mechanical room of the Baltimore College of Dental Surgery, for the session 1848-9.

There has been cavities in teeth prepared and filled at the average expense of 3½ hours time on each, . . . . . . . 247

Of this number, forty were introduced in the teeth of the inferior jaw and of the following classes, namely,

| | | |
|---|---|---|
| Bicuspides, . . . . . | 7 | |
| Molares, . . . . . . | 37 | |
| | —40 | |
| Fillings in the teeth of the superior jaw, . . . . | | 186 |
| Of this number there was introduced in the | | |
| Incisores, . . . . . . . | 72 | |
| Cuspidati, . . . . . . . | 9 | |
| Bicuspides, . . . . . . | 40 | |
| Molares, . . . . . . . | 65 | |
| | —186. | |

Of the whole number of teeth filled, three were excavated and plugged to the apices of the fangs. Seven were filled over exposed nerves: of the latter class of operations, six-seventh were successful.

Teeth separated by the file, for the removal of caries or the excavation of cavities in teeth, . . . . . . . . . . . 96

Teeth and fangs of teeth extracted, . . . . . . 166

Tartar removed from sets of teeth, . . . . . . . 14

Treated cases for diseased gums, . . . . . . . 20

### Dental Mechanism.

| | |
|---|---|
| Whole number of teeth mounted upon metallic plate, . . . | 514 |
| Of these 16 were whole or entire pieces for the superior jaw 14 in number, . . . . . . . . . . . . . | 324 |
| 1 piece for the superior jaw, . . . . . . . . . | 14 |
| 1 partial " " " . . . . . . . . . | 6 |
| 1 plate, 1 tooth retained with clasps for the superior jaw, . | 1 |
| 1 piece of 2 teeth, retained by clasps, . . . . . . . | 2 |
| 2 " 4 " " " . . . . . . . . | 8 |
| 4 " 5 " " " . . . . . . . . | 20 |
| 2 " 6 " " " . . . . . . . . | 12 |
| 1 " 9 " " " . . . . . . . . | 9 |
| 5 " 12 " " " . . . . . . . . | 60 |
| 2 entire pieces for the superior jaw retained by atmospheric pressure with air-chamber, 14 teeth, . . . . . . . . . | 28 |
| 6 pieces retained by atmospheric pressure, 3 teeth each, . . | 18 |
| 2 " " " " 4 " . . . . | 8 |
| 1 " " " " 2 " . . . . | 2 |
| 1 " " " " 1 " . . . . | 1 |
| | 514 |
| Treated cases of irregularity, . . . . . . . . . | 5 |

This being over, Dr. E. B. Gardette, of Philadelphia, arose and pronounced the valedictory—a farewell not soon to be forgotten, as it was replete with the experience of his own very extensive practice—faithfully shadowing forth the shoals and quicksands which each, who had just received the highest honors, must expect to encounter in the practice of his profession; but which obstacles, he very happily showed them, instead of nipping their youthful energies in the bud, and crushing their high resolves and noble purposes—on the contrary, would only prove as useful incentives to loftier and nobler efforts in behalf of their profession—provided they would just bear in mind one thing; and that that one thing, with them, was every thing; namely, the forming a *proper character*—that this was an element of their being, and that it was essential to their permanent success and respectability. The Dr. here very happily drew a broad distinction between reputation and character: that the former was what others thought of us—that it was entirely extrinsic, and that an individual might, in the eyes of the world have a *great reputation*, and still be a villain at heart and in practice. While, on the other hand, character must be made by each one for himself—and on it hung his future destiny, whether for good or evil, as it was in the power of each to form either a good or bad character, and consequently be either a blessing or a curse to mankind. We might be disposed to say much more of the address, but we greatly prefer inviting our readers to a perusal of it for themselves, which we feel assured will be much more acceptable, and which they will find in another part of the Journal.

After Dr. Gardette had concluded, Dr. Philip H. Austen, one of the graduates, on behalf of the class, arose, and feelingly addressed the Faculty and Examining Committee as follows:

*Gentlemen of the Faculty and of the Examining Committee:*

Permit me, on behalf of the graduating class, briefly to respond to the cordial and encouraging words with which you have bid us Godspeed upon our professional career. The life-time of no single individual is sufficient for the attainment of perfection in any science. The present age must build upon the foundation laid by the past, and the succeeding generation continues the unfinished superstructure. Thus is it in every profession; thus, in that one in which we have now become co-workers with yourselves. The time is fresh in the memory of each one of you, when every improvement in dental science was held in closest secrecy by its illiberal discoverer, or else doled out in unsatisfactory portions, at exorbitant charges. How can any science advance under such practice; or any profession become dignified and ennobled while made up of such men!

Fortunately for us—most fortunately for our art—we have fallen upon happier times. We meet, at every turn, noble and liberal minds, ever ready to impart with cheerfulness the result of years of individual toil. This Institution, from which we this night go forth, stands a monument of such disinterested generosity. Two years within these walls have given us a knowledge and a proficiency, to acquire which, cost each of you, gentlemen, many of the best and most vigorous years of your lives. I say not this in a spirit of boasting, but with a feeling of grateful indebtedness to you, our instructors, to whom we owe so much. Because the student of mathematics may, in the brief space of one year, make himself master of those *principia* which cost the life's toil of the immortal Newton, shall he therefore venture to measure himself with that giant mind? It is one thing to discover—it is another, and a far different thing, to understand, apply and profit thereby. The electric telegraph of Morse recalls the memory of Franklin, of Volta and of Galvani; while every improvement made in the steam engine serves but to shed a brighter halo around the names of Watt and Fulton. Yours it has been, gentlemen, to discover—ours to appreciate, apply, improve; yours to lay the foundation and begin the edifice—ours, by untiring industry, to continue the work in the same substantial manner in which it has been begun.

We go forth from this institution under a full sense of the obligations your instructions here have laid upon us. You heard with pride and with pleasure, from the eloquent speaker of this morning,* that wherever,

* Dr. J. H. Foster, of N. Y., in an address before the Society of the "Alumni of the Baltimore College of Dental Surgery."

throughout the south and west, a young graduate of the Baltimore College of Dental Surgery went, he was sure to gain practice—by virtue of this diploma which I hold; let it be ours, classmates, to see that in *our* practice we bring no reproach upon it. Our first aim shall be to advance and do honor to Dental Science; yet never shall we lose sight of our indebtedness to this institution, which enables us to start forth from such high vantage ground. Never, I sincerely trust, shall we become so engrossed in the pursuit of personal schemes, as to turn from any appeal made to us on its behalf, for aid or support.

Whatever may be the changes in life through which we shall be called upon to pass, we shall ever cherish feelings of the highest esteem and regard towards you, our instructors. For your unvarying kindness and courtesy, your unwearied exertions, your generous forgetfulness of self-interest, in the effort to fit us for the right discharge of our coming duties, we feel a debt of gratitude which we wish never to forget. With these feelings of grateful kindness, we now enter upon our career, bearing before us that "banner with a strange device—Excelsior." Gentlemen of the Faculty and of the Examining Committee, in the name of the Graduating Class, I bid you an affectionate Farewell.

After this the benediction was pronounced, and the assembly dismissed, the exercises having passed off apparentiy to the entire satisfaction of all present.

It may be well to state that the ceremonies of the evening were interspersed throughout with appropriate music.

The following comprises a list of the Graduates, with their names, residence, and subject of thesis:

| | NAMES. | RESIDENCE. | THESES. |
|---|---|---|---|
| 1. | Chas. W. Ballard, M. D., | New York, | Physical Indications of the Teeth. |
| 2. | Philip H. Austen, M.D., | Maryland, | Abuse of Mercurial Preparations. |
| 3. | M. A. Hopkinson, | Mass. | Causes and Consequences of Caries of the Teeth. |
| 4. | W. L. Feemster, | Tennessee, | Effects of Diseased Teeth on the General Health. |
| 5. | A. A. Blandy, M. D., | Ohio, | Nervous Disorder. |
| 6. | J. H. A. Fehr, M. D., | Kentucky, | General Dental History. |
| 7. | J. U. L. Warren, | Kentucky, | Medico-Dental Education. |
| 8. | R. R. Sams, | S. C., | Treatise on Caries. |
| 9. | Albion Martin, | Maine, | Extraction of Teeth. |
| 10. | Wm. S. Miller, | Virginia, | Caries of the Teeth. |
| 11. | Jerome Cherry, | Maryland, | Mechanical Dentistry. |
| 12. | George W. Watkins, | Georgia, | Professional Excellence. |
| 13. | Thomas Littig, M. D., | Maryland, | Eruption of the Teeth. |

***Proceedings of the First Annual Meeting of the Society of the Alumni of the Baltimore College of Dental Surgery: furnished for the American Journal and Library of Dental Science.***

*Thursday, March* **1st,** 1849.

The members were called to order at 9 o'clock, A. M.; Dr. H. Colburn in the chair. The minutes of the preliminary meetings were read and adopted. After some debate upon the resolutions laid before the Society by the preliminary meetings, they were passed.

The Society then proceeded to the adoption of the Constitution and By-Laws. On motion of Dr. C. W. Ballard, the Constitution was then signed by the following gentlemen, who became, *de facto,* the founders of the Society, viz. C. W. Ballard, M. D., P. H. Austen, M. D., Hervey Colburn, M. D., J. U. L. Feemster, M. J. Cherry, Robert R. Sams, F. N. Seabury, R. W. Armstrong, L. S. Burridge, T. D. Thompson, C. O. Cone, M. D., A. Martin, G. W. Watkins, M. A. Hopkinson, J. F. Warren, J. H. A. Fehr, M. D., C. A. Barnes.

During the signing of the Constitution, several letters were read before the Society, and the Society then proceeded to the election of the following officers for the ensuing year:

| | |
|---|---|
| Dr. H. Colburn, | *President.* |
| " P. H. Austen, | *Vice-President.* |
| " C. O. Cone, | *Treasurer.* |
| " R. W. Armstrong, | *Corresponding Secretary.* |
| " M. J. Cherry, | *Recording do.* |

*Standing Committee.*—Drs. C. O. Cone, M. A. Hopkinson, and J. U. L. Feemster.

*Committee on Dental Practice.*—Drs. Warren, Fehr and Ballard.

At this time the Society gave way for the Address of Dr. J. H. Foster, of New York City.

After the Address, it was unanimously resolved, that Dr. Foster be presented with a vote of thanks by the Society, and that his address be requested for publication.

Previously, however, to the address, a resolution was offered by Drs. Cone, Colburn and Austen, that the following gentlemen be made honorary members of the Society: Drs. J. H. Foster, of N. Y., E. B. Gardette, Phila., E. Parmly, N. Y., J. Parmly, N. Y., Daniel Harwood, Boston, S. P. Hullihen, Wheeling, Va., E. Townsend, Phila., E. Maynard, Washington City, C. A. Harris, Balt., J. R. W. Dunbar, Balt., W. W. Handy, Balt., J. I. Cohen, Balt., T. E. Bond, Balt., W. R. Handy, Balt.

After the address, another resolution was offered by Drs. Cone, Hopkinson and Colburn, that Dr. Robert Nasmyth, Jr., F. R. C. S., London, be elected an honorary member. These resolutions were passed, and the meeting then adjourned till 3, P. M.

*Three o'clock, P. M.*

Adjourned meeting called to order, Dr. Colburn in the chair. The Society then proceeded to the election of the different committees.

Drs. Austen, Feemster and Hopkinson were elected upon the Committee on Mechanical Dentistry.

The Committee for the Selection of Subjects for Scientific Investigation, viz. Drs. C. O. Cone, Colburn and Fehr.

Committee of Arrangements for the next Annual Meeting, viz. Drs. Armstrong, Cherry and Austen.

Letters were then read from Drs. Wilkes Allen, J. D. Wemple and W. H. Morgan, offering their names for membership. On motion of Drs. Ballard, Seabury and Martin, they were unanimously elected.

The following nominations were then made for the officers for the ensuing year: For President—Dr. Austen and Dr. Fehr; for Vice-President—Dr. Ballard and Dr. Colburn; Treasurer—Dr. C. O. Cone; Recording Secretary—Dr. Ballard and Dr. Colburn; Committee on Practical and Operative Dentistry—Drs. C. W. Ballard, Wilkes Allen and J. D. Wemple—and Drs. Feemster, Seabury and Martin; Committee on Mechanical Dentistry—Drs. Morgan Hopkinson and Warren—and Drs. Armstrong, Cherry and Wemple; Committee on the Selection of Scientific Subjects—Drs. Austen, Feemster and Martin—and Drs. Ballard, Austen and Armstrong.

The following gentlemen were then appointed to deliver dissertations at the next annual meeting—Drs. Austen, Feemster and Fehr.

And the meeting then adjourned *sine die.*

R. W. ARMSTRONG,
*Cor. Sec'y Soc. A. B. C. D. S.*

---

*Cleaveland's Air-chamber Plates.*—The application of artificial teeth, mounted upon a metallic base, without springs or clasps, is regarded by all experienced practitioners as a matter of great importance, and in supplying the loss of a whole denture, or of all of the teeth from one of the jaws, it has been done for the last twelve or fourteen years, upon what is usually termed the atmospheric pressure or suction principle. But, even in cases of this description, considerable difficulty has occasionally been experienced in securing the necessary stability and adhesion of the substitute to the parts against which it is placed, and for the accomplishment of which, various means have been proposed, among these was the construction of the plate or base in such a way as to leave a vacuum or air-chamber between it and the structures against which it was placed. The best description of base of this kind, which we have seen, is the one constructed by Dr. J. A. Cleaveland of Charleston, S. C. about three years ago. The greater portion of the base covering the palatine arch consists of two plates. The upper one has an opening in it about the size of an American twenty-five cent piece, around the edge of which on the lower side, a small half-round wire is soldered. The object of this is to prevent the thin edge of the plate from irritating the mucous membrane, which ultimately adapts itself to the opening in the plate. The lower plate is about one line from the upper.

We have used this description of base in some twelve or fifteen cases, and in every instance with the most satisfactory results. It is necessary, however, that the adaptation of the upper plate to the alveolar ridge and palatine arch, be positively perfect. By using this precaution, a single tooth may be as readily applied as a whole denture.

While upon this subject, we would embrace the opportunity of returning our thanks to Dr. Cleaveland, for the very beautiful air-chamber plate which he had the kindness to send, in the early part of the fall of last year.—*Balt. Ed.*

*Cleaveland's Wax-holders.*—For the procurement of a perfectly accurate adaptation of a metallic base for artificial teeth, it is necessary to have a correct transfer of the parts to which it is to be applied, and to obtain which, an exact impression in wax or some other substance is indispensable. To secure this with the common wax-holder, it is sometimes necessary to take half a dozen impressions. With a view of facilitating this part of the operation, and securing a more accurate result, Dr. Cleaveland of Charleston, S. C., constructed five wax-holders, three for the upper, and two for the lower jaw. The only difference in the upper, is in size, which is varied so as to fit the alveolar border and palatine arch of almost any mouth; the lower have a joint in the centre, so that they may be made to fit any sized jaw—one is intended for taking impressions when four, five or six of the anterior teeth are remaining, and the other where all of the teeth are absent. Messrs. Jones and White of New York, have moulds of these holders and intend manufacturing them for sale. We have the promise of a set, and when we get them we will give a more minute description of them.—*Balt. Ed.*

*Anæsthesia.*—Having been requested by several correspondents, to furnish the readers of the journal with a statement of the effects, which have resulted from the use of ether and chloroform, when administered with a view of producing temporary anæsthesia, and not having leisure, at present to collect all the facts which have been made known, we copy from the Western Journal of Medicine and Surgery, an able article upon the subject, from the pen of Dr. Yandell. All the facts which had transpired upon the subject, at the time of its publication are, we believe, embodied in it. We commend a careful perusal of the paper to our readers.

*The Associated Alumni of the Baltimore College of Dental Surgery.*—This association, as will be seen from the published proceedings, held their first meeting on the first of March, the opening address was delivered by Dr. J. H. Foster of New York; it was appropriate to the oc casion, contains much excellent advice, and was characterized throughout by great ability. We learn that the doctor has been requested to furnish a copy for publication, and we hope to be able to give it to our readers in the next number of the journal. We are sure it will be perused with pleasure by each.

The constitution and by-laws, and the proceedings of the first annual meeting of this society, will be found in another part of the present number.—*Balt. Ed.*

